# Deciphering skeletal muscle development: cellular heterogeneity and molecular regulatory networks from single-cell and spatial transcriptomic perspectives

**DOI:** 10.3389/fcell.2026.1827120

**Published:** 2026-05-22

**Authors:** Xingdong Wang, Yongming Zhang, Huimin Wei, Gerelt Zhao, Ren Mu

**Affiliations:** 1 College of Life Science and Technology, Inner Mongolia Normal University, Hohhot, China; 2 Key Laboratory of Biodiversity Conservation and Sustainable Utilization in Mongolian Plateau for College and University of Inner Mongolia Autonomous Region, Hohhot, China

**Keywords:** animal breeding, cell fate determination, regulatory networks, single-cell RNA sequencing, skeletal muscle development, spatial transcriptomics

## Abstract

The development of skeletal muscle is a biological process of great complexity and high regulation whereby there is a spatiotemporal coordination of interactions amongst various cell and molecular events. Recent advances in scRNA-seq and ST have enabled systematic dissection of skeletal muscle dynamics at single-cell resolution, thereby revealing the underlying molecular regulatory processes. This review provides an overview of existing evidence on molecular components of cellular heterogeneity of skeletal muscle development, which synergistically stabilize transcription factor network, epigenetic regulation, and metabolic reprogramming in cell fate regulation. By systematically comparing findings across species—including humans, mice, pigs, and chickens—we highlight both evolutionarily conserved mechanisms and species-specific regulatory features. This combination of scRNA-seq, ST, and multimodal data enables us to understand the microenvironment as a spatial regulator of muscle stem cell (MuSC) behavior: the composition of the niche, intercellular communication, and mechanical cues. These discoveries not only elucidate the fundamental principles of skeletal muscle development but also provide a theoretical basis for therapeutic strategies against muscle-related diseases and for improving economically important traits in livestock breeding.

## 1 Introduction

Mesodermal somites differentiate to skeletal muscle through a complex series of changes that result in the development of the mature muscle fibers (Tapscott, 2005; Buckingham and Rigby, 2014; Hernández-Hernández et al., 2017). It entails the proliferation, differentiation, migration, and fusion of the myogenic progenitor cells (MPCs) (Lehka and Rędowicz, 2020), and coordination of various cell types in spatiotemporal precision, such as MPCs, muscle satellite cells (MuSCs), fibroblasts, and immune cells (Tierney and Sacco, 2016; Verma et al., 2021). Embryonic MPC genesis and differentiation through postnatal growth, maturation, fiber-type specification and adult muscle homeostasis and repair, multi-layered molecular regulatory networks control every stage of development (Cai et al., 2023b). The skeletal muscle regenerative ability in the postnatal period depends mainly on MuSCs, which are tissue-resident cells (Fu et al., 2015; Fang et al., 2021). The traditional analysis methodologies of bulk tissue or population usually do not provide a measure of cellular heterogeneity and dynamic changes in gene expression (Gulati et al., 2025). The introduction of scRNA-seq and ST can now be used to characterize cellular and spatial tissue organization at a higher resolution than ever before (Zhang et al., 2024).

ScRNA-seq has transformed skeletal muscle development, enabling the objective description of cellular diversity, the development of new subtypes and the mapping of differentiation pathways at single cell scale, which has greatly expanded the understanding of the complex tissue architecture (Tang et al., 2009; Shapiro et al., 2013; Dell’Orso et al., 2019; Giordani et al., 2019). The technology has played a significant role in studying muscle homeostasis and regeneration at different development stages (Walter et al., 2023; 2024). In contrast to conventional surface marker-based technologies, scRNA-seq can discriminate between the subpopulations of MuSCs more precisely, which offers a baseline of understanding of the complexity of functions of such cells in the regeneration process (Byun et al., 2024). Based on this method, scientists have enabled a systematic characterization of developmental stages of skeletal muscle in humans, in the embryonic, fetal, and postnatal stages, and have managed to assemble a spatiotemporal map of skeletal muscle development (Evano et al., 2020).

ST is an effective way of overcoming the loss of spatial information associated with scRNA-seq (Ståhl et al., 2016; Moor and Itzkovitz, 2017). Although scRNA-seq accurately determines cell types, it fails to maintain their native cellular location in undisturbed tissue (Asp et al., 2020). ST, in contrast, fixes gene expression patterns to their initial positioning space, preserving spatial context and what the individual molecules comprising a functional tissue domain entail (Ståhl et al., 2016; Eng et al., 2019; Asp et al., 2020). The method also measures transcriptional status of target cells and surrounding environments which expands and enhances single-cell analyses to multicellular space dynamics and provides new perspectives on tissue architecture in physiological settings (Moncada et al., 2020). Combination of scRNA-seq and ST has become one of the most popular methodologies to study tissues microenvironment, which allows multilayer characterization of cellular properties and tissue functionality. This, together with these datasets, allows researchers to localize cell type-specific transcriptional signatures in a spatial context with accuracy (Heezen et al., 2023). This combined technique has been especially useful in the study of muscles, where it has made possible dissection of the patterns of spatial distributions, and functional heterogeneity among the various different cell types. Such multidimensional integration plans are of immense importance to analytical resolution and the better spatiotemporal characterization of the critical regulation events.

This review aims at discussing how scRNA-seq and ST have been used to decompose cellular and molecular processes in skeletal muscle development and regeneration. We synthesize their contributions to the breakthroughs in the field of revealing the heterogeneity of cells, mapping the spatiotemporal developmental landscapes, and unravelling molecular regulatory networks in a systematized manner. We also explain their general implications to basic biology, clinical medicine and livestock breeding with specific reference to the ways in which these technologies can unravel the molecular and cellular pathways to economically relevant phenotypes in livestock, e.g., muscle growth rate, feed efficiency, lean meat percentage and meat quality characteristics.

## 2 Elucidating muscle development dynamics via scRNA-seq

**FIGURE 1:**
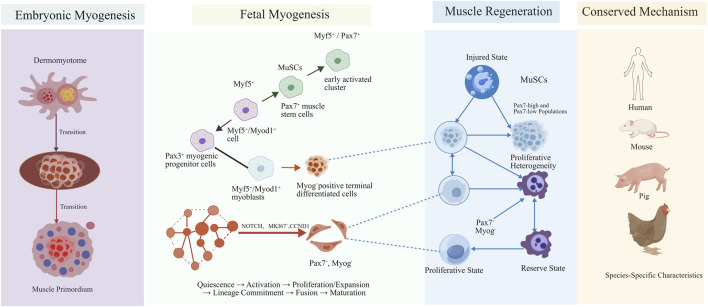
Schematic illustration of the complete process of skeletal muscle development and cell state transition. Note: This figure adopts a timeline design to fully present the three core stages of skeletal muscle from embryonic development to adult regeneration (embryonic period, fetal period, and adult period). The left part of the figure shows the formation process from the dermomyotome to the muscle primordium during the embryonic period; the middle area systematically presents the evolutionary path of the myogenic cell lineage (Pax3^+^ early progenitor cells → Pax7^+^ myogenic progenitor cells → Myf5^+^/Myod1^+^ myoblasts → Myog^+^ terminally differentiated cells), as well as the continuous full trajectory of state transition of muscle stem cells (MuSCs), which follows the sequence of quiescent activation → proliferation and expansion → directed differentiation → fusion and maturation; the right part demonstrates the injury response and regenerative repair process of adult skeletal muscle. Different colors and shapes are applied to distinguish distinct functional subsets of MuSCs, with specific molecular markers of each subset annotated. Meanwhile, key regulatory signaling pathways including NOTCH, MEK/ERK and RXR are displayed, and the conserved regulatory mechanisms and species-specific characteristics of skeletal muscle development in human, mouse, pig and chicken models are compared. The core molecular markers and key signaling pathways involved in this study are labeled at the bottom of the figure. This figure was generated and formatted by BioGDP (https://biogdp.com), and the publication license for academic journals has been obtained (Jiang et al., 2025).

The innovative developments in the scRNA-seq have given the scientists the power to interrogate cellular heterogeneity in muscle growth on an unprecedented scale (Tang et al., 2009). In addition to identifying various cell types that exist in skeletal muscle between embryonic and adult development, the technology has also used it to record the dynamic transcriptional transitions during differentiation (Qiu et al., 2023). Through precise profiling of transcriptomes at the single-cell scale, researchers have been able to identify major subpopulations such as MPCs and MuSCs and make the dynamic evolution of gene expression networks along their differentiation paths (De Micheli et al., 2020; McKellar et al., 2021). Embryonic skeletal muscle originates from the dermomyotome of somites. MPCs are characterized by Pax3^+^ expression, and gradually transition into Pax7^+^ myogenic precursor cells as development proceeds, giving rise to embryonic myotubes and fetal myotubes (Figure 1). Based on single-cell trajectory analysis, embryonic myogenic differentiation follows a highly conserved pathway: Pax3^+^ early progenitors → Pax7^+^ myogenic progenitors → Myf5^+^/Myod1^+^ myoblasts → Myog^+^ terminally differentiated cells (Zhao et al., 2021). In recent years, multiple studies based on scRNA-seq have not only systematically delineated the cellular lineage trajectories of skeletal muscle development in mice and humans, but also identified novel molecular markers and signaling networks that regulate the fate determination of muscle stem cells (MuSCs), providing a fundamental basis for understanding myogenic mechanisms, disease intervention, and livestock and poultry breeding (Barruet et al., 2020).

### 2.1 Identification of MuSCs subpopulations

MuSCs are the core cells responsible for maintaining adult skeletal muscle homeostasis and injury-induced regeneration (Lepper et al., 2011). They reside beneath the basal lamina of muscle fibers and predominantly exist in a quiescent state. Upon injury, MuSCs can rapidly activate, proliferate, differentiate, and self-renew to repair muscle fibers and preserve the stem cell pool (Fu et al., 2022). The application of scRNA-seq and snRNA-seq has overcome the limitations of traditional surface marker-based sorting, enabling unbiased dissection of MuSC heterogeneity. Focusing primarily on adult mouse and human muscle, relevant studies have systematically revealed the functional subsets and molecular characteristics of MuSCs during homeostasis and regeneration.

According to the scRNA-seq results of about 22,000 cells from ten adult donors, the study identified 16 distinct cell clusters, most of which were non-myogenic. Within the MuSC population, two subsets were further distinguished: quiescent and early-activated subpopulations. A study based on large-scale single-cell sequencing of adult human muscle (approximately 22,000 cells) clearly classified MuSCs into quiescent and early-activated subsets. Quiescent MuSCs highly express Pax7, DLK1, and CHRDL2, whereas early-activated MuSCs show sustained Myf5 expression and a gradual downregulation of Pax7 (Fu et al., 2022). These two subsets exhibit significant differences in proliferative potential, differentiation propensity, and self-renewal capacity (De Micheli et al., 2020). As a core regulator of MuSCs, the expression level of Pax7 directly determines cell fate (Bharathy et al., 2013): the high-Pax7 subset possesses strong asymmetric division ability and more stable quiescence maintenance, responsible for self-renewal of the stem cell pool; the low-Pax7 subset is more prone to exit quiescence and initiate differentiation, dominating muscle fiber reconstruction after injury (Bharathy et al., 2013; Fu et al., 2022). This gradient expression pattern serves as a central molecular switch for MuSC fate decision and is highly conserved across mammals, including mice, humans, and pigs (Feng et al., 2023). However, species-specific differences exist: for instance, porcine MuSCs exhibit a broader Pax7 expression range and a more gradual transition to Myf5^+^ states compared to mice, reflecting adaptations to different postnatal growth rates (Qin et al., 2025). snRNA-seq has further classified MuSCs into proliferative and reserve subsets at the functional level. Proliferative MuSCs highly express cell-cycle genes such as MKI67 and CCND1, and are responsible for rapid expansion following injury. Reserve MuSCs exhibit high Pax7 and low Myog expression, retain stem cell properties, and serve as the core cell population for stem cell pool reconstitution after regeneration (Zhang et al., 2024). Mouse injury-regeneration models have confirmed that only quiescent Pax7^+^ MuSCs exist in intact muscle. Upon injury, a heterogeneous cell population encompassing activated, proliferative, and differentiated states rapidly emerges, whose composition and transition dynamics directly determine the efficiency of muscle regeneration (Nahlé et al., 2025).

MuSC heterogeneity exhibits distinct anatomical location specificity, a conclusion validated in both mouse and human studies. MuSCs derived from extraocular muscle (EOM) highly express Pitx2, whereas those from tibialis anterior (TA) muscle preferentially express Pax3. Such region-specific transcriptional signatures constitute the molecular basis for functional adaptation of different muscles, and also lead to inherent differences in regenerative capacity and engraftment efficiency among MuSCs from distinct anatomical sites (Evano et al., 2020; Oprescu et al., 2023). Recent studies have further identified a novel CAV1^+^ MuSC subset characterized by superior engraftment ability and low proliferative activity, making it an ideal seed cell for adult muscle stem cell homeostasis maintenance and cell therapy (Almada and Wagers, 2016). Table 1 provides an overview of major markers expression patterns, functional characteristics, and subpopulation identities of MuSCs.

**TABLE 1:** Muscle stem cell subpopulations and their molecular and functional characteristics.

MuSCs subpopulation	Key marker expression profile	Functional characteristics
Quiescent MuSCs	High Pax7, DLK1, and CHRDL2 (De Micheli et al., 2020)	Maintain stem cell pool homeostasis; strong self-renewal capacity (Rocheteau et al., 2012)
Early-activated MuSCs	Downregulated Pax7 with sustained Myf5 (De Micheli et al., 2020)	Prone to differentiation; short activation latency (Fu et al., 2022)
High-Pax7 MuSCs	Continuous gradient of high Pax7 (Fu et al., 2022)	Strong asymmetric division capacity; prolonged cell cycle entry (Fu et al., 2022)
Low-Pax7 MuSCs	Low Pax7 expression levels (Fu et al., 2022)	Readily activated and initiate differentiation (Rocheteau et al., 2012)
Proliferating MuSCs	High MKI67,CCND1 (Melzener et al., 2024)	Active proliferative capability
Reserve MuSCs	High Pax7, absence of Myog (Melzener et al., 2024)	Retains stem cell identity
EOM-derived MuSCs	Characteristically high Pitx2 (Evano et al., 2020)	Region-specific functionality
TA-derived MuSCs	Preferential Pax3 expression (Evano et al., 2020)	Region-specific functionality
CAV1^+^ MuSCs	Expresses CAV1 (Rocheteau et al., 2012)	Unique engraftment potential and low proliferative activity

### 2.2 Novel cell subpopulation characterization

The quality of scRNA-seq has resulted in new opportunities of discovering new and uncommon populations of cells in the course of muscle development. An example of this method is finding two different fibro-adipogenic progenitor (FAP) subtypes with distinct genomic signatures of human and mouse skeletal muscle samples that had been identified (Micheli et al., 2020). There is also single cell work that indicates that there are many MuSC subpopulations whose fate determination is divergent during regeneration (Rocheteau et al., 2012). The scRNA-seq is highly sensitive, and thus, can identify cell groups that only make a small portion of the overall cellular composition. A publicly available scRNA-seq database of muscle tissues, which combines transcriptomic measurements of approximately 365,000 cells, has now been established, which facilitates the work of researchers in studying transient rare cell types in muscle injury and repair (Jovic et al., 2022).

The endo-pericytes and interstitial tenocytes are two new cell types that have been discovered through the study of human skeletal muscles. Endo-pericytes express markers of endothelial and perivascular cells (Giordani et al., 2019), and may be specialized to play an important role in the regulation of vascular stability and in the muscle microenvironment. The tenocytes of the interstitium specifically express tendon related genes including SCX and TNMD (Giordani et al., 2019), which suggest a potential involvement in myotendinous junction homeostasis and in the repair process, which is commonly injured by sports injuries and degenerative diseases. More exploration of these cells can offer new therapeutic opportunities to the muscle-related diseases. It is noteworthy that a distinctive group of co-expressing myogenic precursor and tenocyte markers in the muscle-tendon interface has been identified, indicating that transitional cell may exist between these two cell types (Liu et al., 2019; Yaseen et al., 2021; Esteves De Lima et al., 2021). Experiments done on transplantation of mouse MuSCs with aging cells showed that the success rate of transplantation diminished two-thirds between aged cells and young cells in the case of transplantation using 10 recently-isolated MuSCs. Nevertheless, this difference was eliminated by the transplanted cells numbering 100–300, which suggests that the heterogeneity of functions occurred in the aged pool of MuSCs (Almada and Wagers, 2016). This observation highlights the dynamic property of transitional cell population at the time of muscle regeneration and the functional changes of that population in relation to age. Recent discoveries in cell subpopulations frequently have stated certain patterns of spatiotemporal distribution, which suggests that the functions can be coupled closely to the microenvironmental cues.

### 2.3 Lineage trajectory reconstruction

Adult MuSC differentiation follows a continuous trajectory: quiescence activation → proliferation and expansion → directed differentiation → fusion and maturation. Using mouse injury-regeneration models and adult human muscle cells, scRNA-seq combined with pseudotime analysis and RNA velocity analysis has reconstructed the continuous cell state transitions and core regulatory axes of this process, establishing a complete logical framework.

#### 2.3.1 Continuous state transitions during adult MuSC differentiation

Upon sensing injury signals, quiescent MuSCs (Pax7^+^, Sdc4^+^) rapidly upregulate *Myod1* and *Islr* to enter an activated state. They subsequently progress to the proliferative phase (Mki67^+^, Top2a^+^) and undergo massive expansion to meet the demands of muscle repair. Finally, they upregulate Myogenin and Myh3, exit the cell cycle, and fuse to form new muscle fibers, thereby completing differentiation (Oprescu et al., 2020). Table 2, along with the expression of the particular marker genes, major activities of the signaling pathway, and the functional features of the characteristic subpopulations (Rocheteau et al., 2012).

**TABLE 2:** Key cellular subpopulations and their molecular signatures during MuSC differentiation.

Differentiation stage	Key subpopulation	Characteristic gene expression	Pathway activity	Functional features
Quiescent state	Pax7ʰ^i^ state	High Pax7	-	Maintains stem cell quiescence (Rocheteau et al., 2012)
Early activation	Sdc4ʰ^i^/Notch2ʰ^i^ state	High Sdc4, Notch2	NOTCH	Key node in myogenic fate determination (Rocheteau et al., 2012; Evano et al., 2020; Micheli et al., 2020; Melzener et al., 2024)
Proliferation phase	Cyclin-enriched State	High cyclin genes	MEK/ERK	Active cell cycling (Melzener et al., 2024)
Differentiation initiation	EM cluster	Co-expression of Myf5 and Myod1	–	Onset of myogenic commitment (Fung et al., 2022)
Terminal differentiation	LM cluster	High Myog, downregulated Myf5	–	Myotube formation stage (Fung et al., 2022)
Maturation stage	Dll1ʰ^i^ state	High Dll1	–	Mature skeletal myocyte identity (Rocheteau et al., 2012)
Cross-species (Daheng broiler)	Myofibrillar cells	Activated SNRPG, SNRPE	–	Mid-to-late differentiation features (Li et al., 2025)
Cross-species (Tibetan chicken)	Myofibrillar cells	High Myog, MYBPH	–	Late-stage differentiation characteristics (Li et al., 2025)
*In* *vitro* model	hPSC-PAX7 cells	Embryonic-to-fetal transition genes	–	Recapitulates natural development (Evano et al., 2020)
Fiber-type transition	Bovine MPCs	Fiber-type marker genes	–	Type I ↔ IIA ↔ IIX transition (Micheli et al., 2020)

#### 2.3.2 Core signaling pathways and regulatory nodes

The core signaling pathways underlying the fate determination of adult MuSCs are highly conserved: NOTCH signaling dominates quiescence maintenance and self-renewal and inhibits differentiation through the NICD-RBPJ axis (Wei et al., 2024; Gao et al., 2025); MEK/ERK signaling regulates proliferative expansion and drives cell cycle progression; and RXR signaling participates in the initiation of differentiation and acts synergistically with Myogenin to promote terminal differentiation. Recent studies have confirmed that the precise temporal activation of these pathways is crucial for balancing stem cell pool maintenance and muscle fiber regeneration, and either excessive activation or inhibition will result in regeneration failure.

#### 2.3.3 Species conservation and human-specific features

The core regulatory axis of adult MuSC differentiation is highly conserved between mice and humans, both following the Pax7→Myf5/Myod→Myogenin transcriptional cascade. However, human MuSCs exhibit slower activation and differentiation kinetics, accompanied by higher expression levels of quiescence maintenance-related genes. Comparative transcriptomic analysis between myogenic progenitors derived from human pluripotent stem cells (PSCs) and naturally developing muscle cells reveals that hPSC-PAX7 cells closely resemble those at the embryonic-to-fetal transition stage (Evano et al., 2020), providing a reliable system for modeling muscle development *in vitro* and standardized seed cells for cell therapy.

RNA velocity analysis has confirmed that pericytes in adult muscle possess multilineage differentiation potential and can differentiate into myogenic progenitors, adipocytes, fibroblasts, and other cell types. As an important reserve cell population, they contribute to microenvironmental remodeling and cellular replenishment following injury, a conclusion validated in both mouse and human tissues (Evano et al., 2020).

However, it is critical to recognize the inherent limitations of pseudotime analysis and RNA velocity when interpreting myogenic lineage progression. These computational approaches generate inferential, model-dependent trajectories rather than direct empirical lineage records. Pseudotime ordering relies on assumptions of continuous, linear differentiation and is sensitive to dropout events, cell cycle artifacts, and heterogeneous sampling depths. RNA velocity, while powerful for predicting directional transitions, is limited by incomplete splicing kinetics modeling, technical noise in unspliced/spliced mRNA quantification, and inability to resolve non-directional or cyclic cell state transitions. Thus, trajectories reconstructed from these methods should be interpreted as hypothetical developmental paths that require validation via orthogonal approaches such as lineage tracing, live imaging, or functional genetic perturbation.

### 2.4 Decoding transcriptional regulatory networks

The spatiotemporal specificity of the transcriptional regulatory network that controls skeletal muscle development is astounding with the basic mechanism being a synergistic interaction process involving myogenic regulatory factors (MRFs) and their regulators (Weintraub et al., 1991). Pax3 and Pax7, which are major early regulators, both mark a primitive myogenic cell population (Relaix et al., 2005). The continued expression of Pax3 and Pax7 following such cells migrating into the myotome indicates that adult muscle MuSCs are lineage-related to embryonic myogenic precursors (Buckingham and Rigby, 2014). In embryogenesis, Pax3 directly regulates the myogenic enhancer of Fgfr4 and regulation of Sprouty genes. At the same time, Pax3 indirectly regulates Myf5 expression by the early epaxial enhancer (EEE). Six homeodomain transcription factors like Six1 and Six4, which are expressed in the dermomyotome, regulate the expression of the downstream myogenic factors directly by targeting enhancers of Myf5 and Myod genes (Buckingham and Rigby, 2014).

Myogenic determination factors Myod and myogenin are differentially regulated in cell cycle during myocyte differentiation. It has been shown that Myod upregulation in the G1 phase facilitates differentiation commitment whereas Myf5 upregulation in G0 phase suppresses differentiation commitment (Zou et al., 2000). This differentiation of regulation can be of the essence with reference to muscular fiber-type transitions. According to the C2C12 differentiation model, Myod mRNA levels do not change much during differentiation with myogenin mRNA levels rising significantly during early differentiation before leveling off (Brown et al., 2012). This dynamic pattern of expression indicates that myogenin could be an important transcriptional regulator of sarcoplasmic reticulum (SR) and contractile protein genes expression. Myogenin is experimentally proven to directly activate the expression of non-muscle cells of SR genes and contractile protein genes (Arai et al., 1992).

There are several important signaling pathways participating in the coordination of cell growth and determination of cell fate. The InR/Akt/TORC1 signaling directly stabilizes the Socs36E protein via its effector complex TORC1 that is also a suppressor of JAK/STAT signaling (Lu et al., 2020). This control system is very well preserved in different species indicating its regulatory ability in all development systems. It is important to note that although the JAK/STAT pathway is an important signaling pathway that defines the fate of follicular cell migration, the InR/Akt/TORC1 and JAK/STAT pathways are antagonistically related during muscle development (Lu et al., 2020).

The Notch signaling pathway is essential in ensuring that muscle stem cells are in the state of quiescence. When notch intracellular domain (NICD) binds to DLL1/4 ligands, it leads to the release of the transcription factor, RBPJ (Rocheteau et al., 2012). In the meantime, the FOXO3 signal pathway also helps the maintenance of muscle stem cell quiescence but its expression decreases slowly with age (Rocheteau et al., 2012). These results highlight the nature of transcriptional regulatory networks, in which the combination of several signaling systems defines cell fate due to highly complex crosstalk. This study creates new understanding of the molecular pathway of muscle growth and highlights the possible therapeutic potential of regenerative medicine and disease treatment. Beyond transcriptional regulation, epigenetic mechanisms also play a pivotal role in modulating myogenic progression.

### 2.5 Epigenetic modification landscapes

The merging of epigenetics information has also increased the dimensions of multi-omics studies. A multi-omics approach on single cells explains the dynamic cross-bridges between transcription factors and regulatory elements on DNA by identifying accessible regions of chromatin (Cai et al., 2023a). The combination of scRNA-seq and scATAC-seq can be used to methodically describe the transcription factor-mediated regulatory events at myogenesis. As an example, a comparative study of scRNA-seq and scATAC-seq in skeletal muscle cells of pigs revealed all major cell types that comprise the myogenesis, rebuilt the developmental pathway of porcine skeletal muscle ontogeny, and identified key transcriptional regulators that drive differentiation, which finally formed a complete transcriptional regulatory network of myogenic progression (Cai S. et al., 2023). The main mechanism of muscle stem cell fate determination is the synergistic combination of chromatin accessibility and transcriptional regulation. It has been shown that in differentiating hEMuSCs to neural progenitor cells (NPCs), 2,438 genes undergo simultaneous alterations in their expression and chromatin accessibility its up/downregulated genes include 1,489 and 949, respectively (Linying et al., 2025). The key aspect of cell fate determination is directly supported by the high correlation of the expression of genes with the openness of the chromatine (R = 0.47, P < 0.001) (Linying et al., 2025). To be more specific, Pax7 in muscle stem cells is associated with chromatin openness, which is achieved by recruiting the Trithorax complex to increase the levels of H3K4me3 modification, and Myod is the sole protein that controls the expression of target genes by interacting with various epigenetic regulators (Fu et al., 2022).

Dynamic remodeling of chromatin accessibility is accompanied by the transition of muscle stem cell states. The EZH1-PRC2 complex maintains cellular quiescence in the quiescent state by non-canonical Notch signaling (Byun et al., 2024). Prostaglandin E2 (PGE2) reorganizes the transcriptional network of muscle stem cells through the epigenetic repression of AP1 binding sites and the epigenetic amplification of regeneration-related regulatory elements CREB and E-box (Wang Y. X. et al., 2025). During the process of activation. Experiments on transplantation also established that EOM MuSCs partially reprogram their epigenomic states to meet new environmental requirements, and alterations in DNA methylation patterns at the enhancer regions have a strong positive correlation with position-specific gene expression (Evano et al., 2020). Epigenetic control of muscle development is exceptionally spatiotemporal specific. The expression of the MYOD protein is triggered by the p38-α/β MAPK pathway by inhibiting RNA-destabilizing protein tristetraprolin (TTP) a post-transcriptional mechanism instead of a transcriptional one (Almada and Wagers, 2016). The opportunities of the research on skeletal muscle development in pigs show that the dynamic variability of gene expression and chromatin accessibility of differentiation pathways of myoblasts are correlated with definite transcription factors activity (Cai et al., 2023a). It is important to note that myogenesis-related genes display an increased chromatin accessibility among lean pig muscle cells, and adipogenic-related genes have increased accessibility among obese pigs (Dell’Orso et al., 2019). This reciprocal epigenetic regulation is not universally observed in other species: in mice, chromatin accessibility changes are more subtle and diet-dependent, while in humans, exercise-induced epigenetic remodeling dominates over genetic background effects. These interspecies differences underscore the need for species-specific epigenetic biomarkers in livestock breeding. This result presents a direct molecular reason as to why the lean meat percentage of pigs is negatively correlated with intramuscular fat (IMF) concentration, and the investigation of chromatin accessibility profiles as epigenetic biomarkers of genomic selection in swine breeding initiatives. RNA velocity analysis presents a new view of studying epigenetic processes and allows predicting the future state of transcription in a single cell by identifying spliced versus unspliced mRNAs (Manno et al., 2018). The genes that were upregulated throughout the differentiation of hEMuSCs to NPCs were also greatly enriched in neural development pathways such as axon guidance (P < 0.001), neuronal migration (P < 0.001), and development of the forebrain (P < 0.001), and changes that are directly associated with similar changes in chromatin accessibility (Gulati et al., 2025). The aging of muscle research shows that Lamin A/C conformational changes cause epigenetic modifications to cause functional deterioration, which has high fast-twitch fibers and slow-twitch fibers, with a loss of muscle stem cells and the formation of specialized types of protective fibers (Xiong et al., 2020).

Epigenetically speaking, the epigenetic landscape provides a new angle of analysis concerning the complex traits. Epigenetic marks are dynamic in contrast to fixed DNA sequences and can be modified by environmental factors, including nutrition and management (Fang et al., 2025). The analysis of scRNA-seq and scATAC-seq analyses of the development of epigenetic regulation of myogenesis and adipogenesis in livestock (e.g., cattle, sheep, and poultry) would help determine the epigenetic biomarkers of ideal growth and meat quality. An example would be the methylation of a well-established regulator of muscle mass (Laere et al., 2003). Equally, accessibility of chromatin at the MYOD1 locus in cattle might be used as a surrogate of myogenic competence (Afonso et al., 2025). This opens up the possibilities of using epigenetic selection or epigenetic breeding strategies, in which the reduction of animals on the basis of desirable epigenetic phenotypes (e.g., methylation of IGF2 in pigs) may be used to complement genomic selection and speed up the acquisition of genetic gain in complex traits.

## 3 Spatial architectural patterning

The advent of ST has provided new opportunities in the research of the three-dimensional molecular organization of muscle tissue (Wang H. et al., 2025). This technology, which combines spatial coordinates with gene expression patterns, allows to precisely localize the specific cell types in muscle tissue and clarify how the dynamic interactions of these spatial distribution patterns and the local microenvironment are related (Min et al., 2025).

### 3.1 ST methodologies

ST represents a swiftly developing research tool, and its new essence is tied to the fact that it combines the data on gene expression with spatial data to provide *in situ* analysis of RNA molecules in the context of their native tissues (Ståhl et al., 2016). For skeletal muscle research, the core metrics for evaluating spatial transcriptomics technology are: whether its resolution/spot diameter can cover the MuSC niche (approximately 10–30 μm), whether it can distinguish mononuclear MuSCs from multinucleated muscle fibers, and whether it can capture local microenvironmental signals in the muscle fiber repair zone. Among sequencing-based ST technologies, Visium stands out as the most successful in terms of its ability to analyze the spatial distribution of hundreds or thousands of RNA species at subcellular resolution simultaneously (Ståhl et al., 2016). MERFISH belongs to the category of the most successful imaging-based ST technologies that allow to study the spatial distribution of hundreds or thousands of RNA species at subcellular levels at the same time (Chen et al., 2015; Xia et al., 2019). This method is especially effective to examine pathological characteristics of relatively low prevalence in patient cells (myocytes) since it allows examining transcriptomic profiles of both mono and multi-nucleated cells at the same time. In practice, the researchers used a 140-gene probe set, which includes 24 known DUX4 target genes and other disease-related genes, and the Cellpose software to identify the single-nucleus and nuclear segmentation and obtained the accurate resolution of the heterogeneity of gene expression in muscle fibres (Chen et al., 2024). The 10x Genomics Visium is a cutting-edge technology used in sequencing-based ST domain. The Visium slide is spatially barcoded with 5,000 spots (polydT-VN tails) in each capture area to capture 3′ ends of mRNA molecules (Ruoss et al., 2022). Visium platform has shown an exceptional stability in data, with research findings showing that the sequencing capabilities of the platform are not compromised by storage times up to 6 years. TOMO-seq is another spatial sequencing technology that provides a complete view of gene expression patterns on the full muscle tissue by cryosectioning along the anteroposterior axis and then extracting and sequencing any RNA (Mir et al., 2024).

The Stereo-seq technology independently developed by BGI is a recent advancement in ST, involving the use of DNA nanoballs to create sequencing chip-based technology with a 500 nm resolution, and covering capture areas of 13 cm × 13 cm. This platform has been optimally used in the study of plant tissue by employing optimized cryosectioning protocols and permeabilization procedures. Practically, 10 μm-thick cryosections are put on 100 mm^2^ chips designed with a specific purpose, and permeabilization buffer is applied through ultrasonic nebulization to reduce mRNA diffusion. Stereo-seq has also been used to discover 41 different anatomical structures in muscle research applications, and the expression patterns of known marker genes are highly consistent with *in situ* hybridization data (Chen et al., 2022).

Table 3 in a recapitulation of the molecular properties and subpopulation distributions in specific functional regions throughout the skeletal muscle tissue with specific signature gene expression patterns that correlate with specific biological processes, such as muscle regeneration, fibrosis, and calcification.

**TABLE 3:** Region-specific gene expression and functional characteristics in skeletal muscle tissue.

Tissue region	Signature gene expression	Cellular subpopulation features	Tissue region
Muscle regeneration zone	High *Myl4*, *Sparc*, *Hspg2*	Enriched myogenic precursors	Muscle regeneration and repair (Heezen et al., 2023)
Fibrosis zone	Upregulated *Vim*, *Fn1*, *Thbs4*	Dominated by FAPs	Fibrotic progression (Heezen et al., 2023)
Calcification zone	Enriched *Bgn*, *Ctsk*, *Spp1*	Accumulated stromal cells	Tissue calcification (Heezen et al., 2023)
Extraocular muscle (EOM)	Differential *Hox* family expression	Phenotypic switch post-MuSC transplantation	Position-dependent reprogramming (Evano et al., 2020)
Upper limb muscle	Specific *PITX1* expression	Anatomically distinct subpopulations	Limb patterning (Gulati et al., 2025)
Lower limb muscle	Distinct *Hox* gene profiles	Spatially restricted subpopulations	Functional specialization (Evano et al., 2020; Gulati et al., 2025)
Injury region (Early)	*Ccl7*, *Cxcl5*, *Cxcl1*	FAPs recruiting immune cells	Inflammatory response (Micheli et al., 2020)
Repair region (Late)	*Postn*, *Bgn*, *Sparc*	FAPs expressing ECM genes	Extracellular matrix remodeling

Table 4 gives an overview comparison of mainstream ST technologies, such as MERFISH, Visium, TOMOseq, Stereo-seq, and microarray based methodologies and details in table format about the various resolutions, throughputs, capture areas, and their main features, to assist researchers in making appropriate choices about the methodologies to use.

**TABLE 4:** Comparison of ST technologies.

Technology name	Technology type	Resolution	Throughput (gene count)	Capture area	Key features
MERFISH	Imaging-based	Subcellular	140–thousands of genes	Single cell/myofiber	Enables simultaneous analysis of mononucleated and multinucleated myotubes; suitable for low-prevalence pathological features (Chen et al., 2015)
Visium	Sequencing-based	100 µm	Whole transcriptome	6.5 × 6.5 mm	Each capture area contains 5,000 spatially barcoded spots; data stability unaffected by 6-year storage (Ståhl et al., 2016)
TOMOseq	Sequencing-based	Tissue-level	Whole transcriptome	Whole muscle tissue	Spatial profiling via cryosectioning along the anteroposterior muscle axis (Mir et al., 2024)
Stereo-seq	Sequencing-based	500 nm	Whole transcriptome	13 × 13 cm	Utilizes DNA nanoball sequencing chips; optimized cryosectioning and permeabilization; identifies 41 anatomical structures (Chen et al., 2022)
Microarray-based methods	Sequencing-based	100 µm	Whole transcriptome	Multiple parallel sections	Capable of processing numerous 100 µm tissue sections simultaneously, though resolution is limited (Giacomello and Lundeberg, 2018)

### 3.2 Spatiotemporal developmental dynamics

The epigenetic modification landscape plays a critical dynamic regulatory role during skeletal muscle development, and its synergistic interplay with the transcriptional regulatory network forms the core basis of cell fate determination. By integrating multi-omics technologies such as scRNA-seq and scATAC-seq, researchers can systematically delineate transcription factor-mediated regulatory events and reveal the precise spatiotemporal coupling between chromatin accessibility changes and gene expression.

During muscle stem cell fate commitment, chromatin accessibility and gene expression exhibit a high degree of coordination. Taking the differentiation of mouse embryonic muscle stem cells into neural progenitor cells as an example, 2,438 genes undergo simultaneous changes in expression levels and chromatin states, among which 1,489 genes are upregulated and 949 downregulated, with gene expression significantly correlated with chromatin openness (R = 0.47, *P* < 0.001). Pax7 recruits the Trithorax complex to increase H3K4me3 modification and promote chromatin opening at specific gene regions (Dong and Cheung, 2021); Myod, as the only protein capable of interacting with multiple epigenetic regulatory factors, directly controls target gene expression. The transition of muscle stem cell states is accompanied by dynamic remodeling of chromatin accessibility—for instance, the EZH1-PRC2 complex maintains quiescence through non-canonical Notch signaling, while prostaglandin E2 reorganizes the transcriptional network via epigenetic regulation to facilitate activation (Vicente-García et al., 2022).

There are notable differences in epigenetic regulatory patterns across species, offering important clues for understanding the mechanisms of muscle development. In mouse models, chromatin accessibility changes are relatively subtle and highly dependent on dietary factors; in human muscle, exercise-induced epigenetic remodeling dominates, overriding the effect of genetic background; studies in pigs further reveal that myogenesis-related genes exhibit enhanced chromatin openness in lean-type muscle, whereas adipogenesis-related genes are more open in obese pigs (Miao et al., 2021). These findings not only explain the negative correlation between lean meat percentage and intramuscular fat content in pigs but also provide a molecular basis for using chromatin accessibility signatures as breeding markers (Gundersen and Anas, 2025).

RNA velocity analysis offers a new approach for predicting epigenetic processes by distinguishing spliced and unspliced mRNAs. During stem cell differentiation into mature cells, upregulated genes are enriched in neural development pathways, and these changes are directly associated with alterations in chromatin accessibility. Aging research has confirmed that conformational changes in lamin A/C trigger epigenetic modifications, leading to decline in muscle function, manifested as an imbalance in fast-twitch and slow-twitch fiber ratios and stem cell loss (Corbett et al., 2023).

Epigenetic marks hold broad promise as biomarkers for complex traits. For instance, the methylation status of the IGF2 gene can serve as an epigenetic indicator that complements genomic selection, while chromatin accessibility at the MYOD1 locus in cattle can reflect myogenic capacity (Li et al., 2022). By analyzing the epigenetic characteristics of myogenic and adipogenic developmental processes across different mammals, it is possible to develop selection strategies targeting growth rate, muscle composition, and meat quality traits, thereby providing a new molecular framework for livestock production. Compartmentalization of Tissue Domains: Implications for Muscle Stem Cell Niches and Myonuclear Heterogeneity.

Skeletal muscle formation entails the differentiation of various myogenic precursor cells at definite anatomy sites, which is very pronounced in the formation of the trunk muscles. Epaxial and hypaxial muscles have different progenitors and tissue microenvironment controls them differently. During skeletal muscle development, two core phases occur sequentially: primary myogenesis (embryonic stage) and secondary myogenesis (fetal stage). Primary myogenesis mainly forms early myotubes and establishes the basic spatial architecture of muscle, whereas secondary myogenesis accounts for a massive increase in the number of muscle fibers, directly determining postnatal muscle mass and total myofiber number in animals. The timing windows of primary and secondary myogenesis differ significantly across species. This interspecies variation is critical for translating findings from model organisms to livestock and humans. In mice, primary myogenesis occurs mainly at embryonic days 10.5–12.5, and secondary myogenesis is concentrated at embryonic days 14.5–16.5 (Biressi et al., 2007). In pigs, primary myogenesis takes place at days 30–35 of gestation, and secondary myogenesis spans days 35–91, during which the myofiber type composition (such as MYH1 and MYH7) and spatial distribution undergo dramatic dynamic remodeling. In humans, primary myogenesis corresponds to the first 4–6 weeks of embryonic development, and secondary myogenesis occurs at weeks 8–14 (Biressi et al., 2007). The same results were confirmed by Visium platform analysis, which showed stereotypic spatial patterning of fast type IIx (MYH1) and slow type I (MYH7) fibers in healthy rotator cuff muscles and injury repair areas had a specific transcriptional pattern linked to degeneration and regeneration (Ruoss et al., 2022). While the previous sections focused on the heterogeneity of MuSCs, it is critical to recognize that the post-mitotic myofibers they form and maintain are not uniform structures. Different myofiber types create distinct niche microenvironments that reciprocally influence MuSC behavior. For instance, slow-twitch (Type I) fibers are associated with a more oxidative niche, while fast-twitch (Type II) fibers reside in a glycolytic niche, and these metabolic differences can profoundly impact MuSC quiescence, activation, and regenerative potential (Relaix et al., 2021).

These patterns of the spatial distribution of the types of muscle fibers are highly connected with their physiological functions. Type I fibres, highly mitochondrion-rich and of high oxidative enzyme activity, have slow and protracted contraction properties. On the contrary, Type IIb fibers are rich in glycogen and show high levels of ATPase activity, and they mainly facilitate rapid, but temporary contractions like anaerobic ones (Biressi et al., 2007). There is ST data demonstrating that the expression pattern of the MYH1 (Type IIx) and MYH7 (Type I) genes also has an evidently mutually exclusive distribution, and this expression signature exhibits a large degree of location-specific variation between the muscles across different anatomical sites (Ruoss et al., 2022). In the process of muscle injury repair, the local regulation of molecular markers, including embryonic myosin heavy chain (MYH8), early embryonic myosin heavy chain (MYH3) and myosin light chain 4 (MYL4) in the regenerating regions is a key source of information on the processes of muscle regeneration (Ruoss et al., 2022). Extending this concept of heterogeneity, recent snRNA-seq studies have revealed that myonuclei within a single multinucleated myofiber are not transcriptionally identical. Multiple myonuclear subtypes have been identified, including those specialized for neuromuscular and myotendinous junction maintenance, as well as a subtype enriched for secretory factors that may modulate the local MuSC niche (Petrany et al., 2020). These studies demonstrate that the muscle fiber itself is a spatially complex syncytium, with distinct nuclear populations at different subcellular domains (e.g., synaptic, perisynaptic, and central nuclei). The application of spatial transcriptomics is beginning to map the precise localization of these myonuclear subtypes and their spatial relationship with quiescent and activated MuSCs, providing a more complete picture of the muscle tissue architecture (Mir et al., 2024).

The use of MERFISH technology has gone a long way in promoting our knowledge of spatial heterogeneity of gene expression during pathological conditions. DUX4 target genes show abnormally high expression patterns in muscle tissues in patients with facioscapulohumeral muscular dystrophy (FSHD), and these spatially heterogeneous patterns of expression suggest that these may play a key role in the pathogenesis of the disease (Chen et al., 2024). The development of skeletal myogenesis in human embryonic limbs has also been found to occur in two phases, each of which is unique in its cellular state and spatiotemporally regulated gene expression program (Wang H. et al., 2025). These results have not only shown the critical importance of ST technologies in the interpretation of skeletal muscle development and disease processes, but also have given new research paradigms on how tissue regionalization and functional specialization are related. Table 5 systematically summarises fiber type-specific marker genes in skeletal muscle providing some important information on their spatial distribution, functional properties, and developmental/pathological correlations. Differential expression patterns of these molecular marker provide valuable information on the heterogeneity of muscle tissue, regeneration and pathogenesis of muscular dystrophies.

**TABLE 5:** Skeletal muscle fiber-type marker genes and their expression characteristics.

Gene marker	Expression regionality	Fiber type/Function	Associated stage/Pathology	Technical validation
MYH1 (Type IIx)	Specific regions in healthy rotator cuff	Fast-twitch fiber; high glycogen content; marked ATPase activity	Dynamic changes in porcine skeletal muscle (E35–E91) (Biressi et al., 2007)	Visium ST (Ruoss et al., 2022)
MYH7 (Type I)	Mutually exclusive distribution with MYH1	Slow-twitch fiber; mitochondria-rich; high oxidative metabolism	Porcine embryonic development (Biressi et al., 2007)	Visium ST (Ruoss et al., 2022)
MYH8 (Embryonic)	Regenerating areas post muscle injury	Embryonic myosin heavy chain; regeneration marker	Muscle regeneration (Ruoss et al., 2022)	Visium ST (Ruoss et al., 2022)
MYL4	Muscle injury repair zones	Myosin light chain; regeneration-associated	Muscle regeneration (Ruoss et al., 2022)	Visium ST (Ruoss et al., 2022)
DUX4 target genes	Aberrantly expressed regions in FSHD patients	Pathology-associated genes; dysregulated in muscular dystrophy	Facioscapulohumeral muscular dystrophy	MERFISH (Chen et al., 2024)

### 3.3 Microenvironmental regulation of MuSC behavior across development and regeneration

**FIGURE 2:**
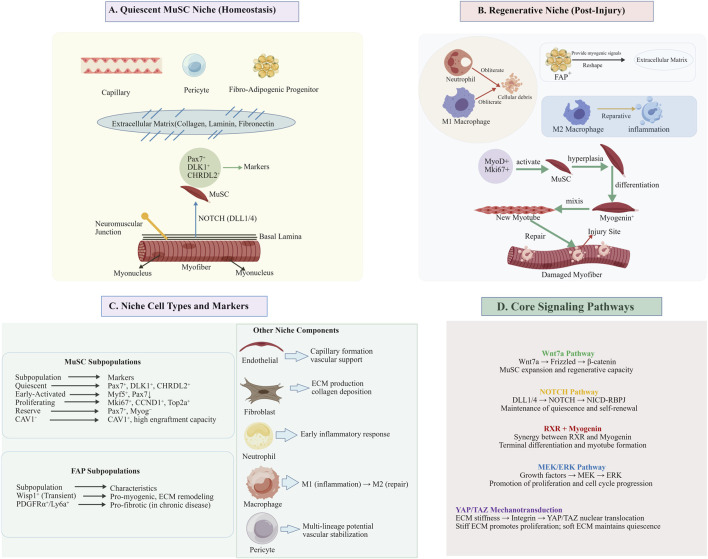
Muscle stem cell niche architecture and cellular interactions. Note: **(A)** In homeostasis, quiescent MuSCs (Pax7^+^, DLK1^+^, CHRDL2^+^) reside between the myofiber sarcolemma and basal lamina, maintained by NOTCH signaling (DLL1/4→NICD-RBPJ) and ECM components (Collagen IV, Laminin, Fibronectin). Neighboring cells include capillary endothelial cells, pericytes, FAPs, and fibroblasts. **(B)** Upon injury, the niche transitions to a regenerative state: neutrophils and M1 macrophages clear debris; MuSCs activate (MyoD^+^, Mki67^+^) and proliferate; FAPs (Wisp1^+^ subset) provide pro-myogenic signals (Mdk) and ECM remodeling; M2 macrophages support resolution; activated MuSCs differentiate (Myogenin^+^) and fuse into new myotubes. **(C)** Niche cellular diversity: functionally distinct MuSC subpopulations (quiescent, early-activated, proliferating, reserve, CAV1^+^) and FAP subpopulations (Wisp1^+^ pro-myogenic vs. PDGFRα^+^/Ly6a^+^ fibrogenic), together with endothelial cells, pericytes, macrophages, neutrophils, and fibroblasts, collectively regulate muscle homeostasis and regeneration. **(D)** Core signaling pathways governing MuSC fate: NOTCH maintains quiescence; MEK/ERK drives proliferation; Wnt7a promotes expansion; YAP/TAZ transduces ECM stiffness cues; RXR synergizes with Myogenin for terminal differentiation. During skeletal muscle development, microenvironmental signals coordinate cell fate determination and heterogeneity maintenance through complex regulatory mechanisms. The Notch signaling pathway promotes SC self-renewal via contact-dependent interactions, while FGF signaling regulates the proliferation and differentiation of MPCs through the MAPK cascade (Grand and Rudnicki, 2007). However, the composition and regulatory logic of the MuSC microenvironment differ significantly between development and regeneration. This figure was generated and formatted by BioGDP (https://biogdp.com), and the publication license for academic journals has been obtained (Jiang et al., 2025).

During skeletal muscle development, microenvironmental signals coordinate cell fate determination and heterogeneity maintenance through complex regulatory mechanisms (Figure 2). The Notch signaling pathway promotes SC self-renewal via contact-dependent interactions, while FGF signaling regulates the proliferation and differentiation of MPCs through the MAPK cascade (Grand and Rudnicki, 2007). However, the composition and regulatory logic of the MuSC microenvironment differ significantly between development and regeneration. During embryonic and fetal development, the niche is primarily composed of other myogenic progenitors, nascent myofibers, and migrating cells from the dermomyotome, with a strong emphasis on directed migration and patterning (Loreti and Sacco, 2022). In contrast, the adult regenerative niche is defined by a robust inflammatory response, followed by dynamic interactions with fibro-adipogenic progenitors (FAPs), endothelial cells, and immune cells (Wei et al., 2021). ST studies have revealed an intriguing phenomenon during muscle regeneration: FAPs high express Mdk, while its receptor genes (Ncl, Sdc4, Lrp1) are significantly enriched in various myogenic cell types. This suggests a conserved, reciprocal signaling axis where FAPs support myogenic differentiation. Recent scRNA-seq has resolved this further, identifying specific FAP subpopulations, such as a Wisp1+ subpopulation that is transiently activated early after injury and is a key source of pro-myogenic and ECM-remodeling signals (Wei et al., 2021). Conversely, a PDGFRα+/Ly6a+ FAP subpopulation has been implicated in supporting fibrogenesis in chronic muscle diseases, highlighting the context-dependent roles of niche cells (Theret et al., 2021). Such spatially and temporally localized ligand-receptor complexes give new insights into microenvironmental control (McKellar et al., 2021).

ECM, being an important element of the microenvironment, has tightly arranged structures in the formation of myofibers. In the embryo, myogenic cells move to certain patterns to establish multinucleated myofibers which are enclosed by a basal lamina made up of type IV collagen and laminin and linked together by fibronectin-rich interstitial connective tissues (Knudsen, 1990). The ECM is not a mere mechanical scaffold, but it also controls the behavior of cells, in which case integrin-mediated signaling is involved. The composition and rigidity of the ECM are potent regulators of MuSC fate. For instance, a stiff ECM, characteristic of fibrotic tissue, promotes YAP/TAZ signaling, driving MuSC proliferation at the expense of self-renewal and contributing to regenerative failure (Tiskratok et al., 2025). In contrast, a compliant ECM mimicking healthy muscle supports quiescence and efficient repair. It is remarkable that the selective upregulation of ECM genes like Col8a1 and Col12a1 in the Wisp1^+^ FAP subpopulation is an indicator that these genes play a significant role in the tissue remodeling in the post-injury period of 3.5–5 days (Oprescu et al., 2020). Reconstructions of co-expression networks of ECM genes in facioscapulohumeral muscular dystrophy patients reveal that alterations in ECM networks are linked to numerous muscular disorders (Chen et al., 2024).

Balanced regulation of transcription factor networks is one of the major ways in which the microenvironment mediates cell fate. In somite formation, the antagonistic interaction between Pax3 and Foxc2 defines the lineage commitments of cells to either myogenic or non-myogenic differentiation (Lagha et al., 2009). On the same note, BMP and Notch signaling pathways induction in the chicken embryonic dermomyotome, respectively, enhances vascular and myogenic fate options (Ben-Yair and Kalcheim, 2005). The experiments of single-cell lineage tracing have established that even cells of the same precursor can take on different cell types under local microenvironmental differences, a vivid example of how the microenvironment is at the core of the regulation of cellular heterogeneity (Tierney and Sacco, 2016).

## 4 Multimodal data integration analysis to decipher muscle development

Effective integration of scRNA-seq and ST requires a multi-step analytical framework that addresses their complementary strengths and weaknesses. Typically, integration proceeds via three core strategies: reference mapping, which annotates cell types from scRNA-seq and projects them onto ST slides to deconvolve spatial spots; joint embedding, which employs algorithms including Harmony, LIGER, or Seurat V5 to align scRNA-seq and ST datasets within a shared low-dimensional space (Miller et al., 2022); and spatial network modeling, which reconstructs ligand–receptor pairs and cell–cell communication by combining spatial coordinates with single-cell expression profiles. Together, these strategies enable systematic mapping of cell identities, spatial domains, and regulatory microenvironments in skeletal muscle (Longo et al., 2021).

The combination of scRNA-seq and ST technologies has put forward new perspectives into exploring the molecular mechanisms of muscle development. This integration is not merely additive but synergistic, as it allows researchers to map the transcriptomic states of individual cells onto their precise spatial coordinates within the tissue, thereby inferring cell-cell communication networks and spatial niches that regulate cell fate. Through single-cell and spatial transcriptomic data integration, Zhang et al. created a spatiotemporal single-cell transcriptomic map of human embryonic skeletal myogenesis development defining two phases with distinct cellular states and cellular gene expression programs (Zhang et al., 2024). This study exemplifies how multimodal data can resolve the timing and location of key developmental decisions, such as the specification of limb muscle progenitors. Although scRNA-seq and snRNA-seq allow capturing whole transcriptomes precisely at the level of single cells, and efficiently distinguishing molecular characteristics of a variety of cells in muscle tissue (Sun et al., 2020; Coulis et al., 2023), the scATAC-seq can provide complementary data on the dynamics of chromatin accessibility in an epigenetic context (Mao et al., 2022; Guo et al., 2025). ST platforms encode information on tissue architecture: Visium and MERFISH, enable the specific localization of gene expression patterns in their original contexts (Sarfatis et al., 2025; Wang H. et al., 2025). This multidimensional data integration plan goes beyond the constraints of the traditional methods to provide new avenues of understanding the cell fate determination mechanisms in muscle development (Coulis et al., 2023).

Most recent innovations in computational models have achieved important new contributions to the analysis of ST and scRNA-seq data, especially in dimensionality reduction, trajectory inference, and network reconstruction (Yang et al., 2024; Klein et al., 2025). Specifically for muscle research, these tools are being adapted to handle the unique challenge of multinucleated myofibers and the high degree of cellular heterogeneity. The new approach called Genes (GCNG) uses graph convolutional networks to convert spatial data into graph form and jointly learns them with gene expression data to effectively discover various new ligand-receptor interactions (Yuan and Bar-Joseph, 2020). Recent advances in the methods of data integration have enabled the comparability of multi-source data sets to significantly increase. The algorithm called Harmony, which is characterized by its computational efficiency and integration performance has become a commodity when it comes to the processing of heterogeneous scRNA-seq data. A massive experiment that involved 23 new samples and 88 public datasets proved that Harmony is more effective at conserving the intrinsic structure of the reduced-dimensional spaces, specifically, it is much more effective at reducing the batch effects due to the various experimental conditions and technical platforms (McKellar et al., 2021).

Significant breakthroughs have also been achieved in ST data analysis methodologies. The Banksy algorithm significantly enhances tissue architecture resolution by integrating cellular gene expression profiles with features from their spatial neighborhoods (Xing et al., 2022). Particularly adapted for the 2 μm-resolution data of the Visium HD platform, this method effectively addresses the limitation of conventional clustering approaches that overlook spatial information. The development of developmental trajectory inference methods has provided new perspectives for understanding myogenesis. The DEAPLOG algorithm, developed by Zhang Hongbo’s team, enables precise localization of dynamic gene expression changes along cellular differentiation paths (Zhang et al., 2024). Concurrently, researchers from the University of California, Los Angeles constructed gene regulatory networks of muscle progenitors and stem cells, confirming that hPSC-derived Pax7^+^ myogenic progenitors closely resemble natural embryonic-to-fetal transitional cells (Evano et al., 2020), establishing a pivotal reference standard for *in vitro* muscle differentiation studies.

Multi-omics integration technologies are advancing toward higher precision and larger scales. The iSCALE method achieves tissue-wide reconstruction of gene expression landscapes by integrating multiple sub-capture regions from Visium platforms (Hallou et al., 2025). This innovation is particularly promising for studying large muscle groups or entire developing limbs, as it enables cellular-resolution analysis of large tissue specimens while transforming conventional H&E-stained images into high-value biological data, significantly reducing the cost barriers for ST studies. In muscle disease research, this technology has been successfully applied to analyze brain tissues from multiple sclerosis patients, demonstrating its clinical applicability. These methodological advances mark a new era in muscle development and regeneration research, enabling simultaneous dissection of spatiotemporal dynamics with unprecedented resolution.

Despite rapid progress, multi-modal integration faces substantial limitations that must be acknowledged. First, technical discrepancies exist in gene capture efficiency, dynamic range, and resolution between scRNA-seq and ST platforms, leading to batch effects that are difficult to fully eliminate. Second, current deconvolution algorithms struggle with multinucleated myofibers, a unique structural feature of skeletal muscle that causes mismatches between mononuclear scRNA-seq profiles and ST signals. Third, spatial data sparsity and high noise reduce accuracy in identifying rare cell types and weak intercellular interactions. Finally, most integration pipelines lack standardization, complicating cross-study comparison and reproducibility. Addressing these challenges will require improved experimental protocols, better computational models, and validated benchmark datasets for muscle tissue.

## 5 Developmental anomalies and disease mechanisms

Abnormal skeletal muscle development is closely associated with various pathological conditions, which exhibit complex molecular mechanisms and cellular heterogeneity. Muscular dystrophies represent a group of inherited disorders characterized by progressive muscle degeneration and impaired regeneration, involving multiple molecular pathways and cellular abnormalities. Duchenne muscular dystrophy (DMD), the most prevalent form, results from mutations in the DMD gene leading to complete absence of dystrophin protein, consequently causing sarcolemmal instability and chronic muscle damage (Crist, 2016). The mdx mouse, a classical DMD model, carries a point mutation in exon 23 of the dystrophin gene generating a premature stop codon that disrupts dystrophin-glycoprotein complex (DGC) function (CHARGÉ and RUDNICKI, 2004). This defect renders muscle fibers more susceptible to contraction-induced injury. Although phenotypically normal at birth, mdx mice develop significant muscle degeneration at 3–5 weeks of age accompanied by transient muscle hypertrophy. With aging, although muscle degeneration/regeneration cycles persist, their efficiency gradually declines, ultimately leading to regenerative failure in 15-month-old mice (Chargé and Rudnicki, 2004). Recent single-cell spatiotemporal atlas analyses have revealed that PITX1 plays a central regulatory role in upper and lower limb skeletal muscle patterning, whose dysregulation directly causes morphological alterations such as brachydactyly or polydactyly (Zhang et al., 2024). Cryo-electron microscopy structural analyses further confirm that conformational changes in Lamin A/C represent key molecular events underlying age-related muscle functional decline, providing novel perspectives for understanding the pathogenesis of aging-associated muscle disorders (Lai et al., 2024).

## 6 Clinical and agricultural translational research

The synergistic development of scRNA-seq and ST technologies has fully deciphered the three-dimensional information of gene expression encompassing “cell type–spatial location–functional state.” This technological paradigm is now driving progress in translational medicine and precision agriculture, spanning both fundamental discovery/clinical diagnostics and agricultural breeding.

### 6.1 Clinical applications and translational research

The single-cell and spatial technologies central to this review are not just basic research tools; they are actively driving translational breakthroughs in muscle disease diagnosis, prognosis, and therapy. Harvard University has created the SkMo culture system, which has a technical breakthrough of 73% ± 9% efficiency in dedifferentiation of myoblasts. The transplanted artificial MuSCs produced a particular force of 138 ± 20.49 kN·m^-2^ (Lai et al., 2024). Not only does this system help to solve the critical problem of cell sourcing, to clinical therapy, which results in up to 1 × 10^7^ therapeutic cells per biopsy; however, when used with CRISPR gene editing technology, it improves the genetic correction rate in hereditary muscular dystrophies by approximately a 12%–89% (Price et al., 2024). This demonstrates a direct translational pipeline where scRNA-seq is used to identify the optimal cell state for transplantation, and gene editing is used to correct the underlying genetic defect.

In the context of aging, a major risk factor for muscle dysfunction, scRNA-seq has pinpointed the cellular mechanisms of decline. The BGI Research team studies have revealed the important changes that occur in the process of aging of muscle stem cells. ScRNA-seq analysis has shown that the number of muscle stem cells has decreased by 62% in old age relative to young adults and that there is an increase in the proportion of FOS^+^ primed stem cells resulting in a 34% decrease in proliferative capability (Lai et al., 2024). The results provide novel insights into the functional aging process, specifically, this is the largest increase in muscle regeneration rate achieved through the delivery of the Wnt7a factor via the AAV that could be used as a clinical intervention (Fang et al., 2021; Nayak et al., 2021).

The patterns of senescence markers during muscle regeneration have been explained as dynamic using scRNA-seq studies. The scientists discovered a unique subpopulation of myogenic stem cells with senescence properties in aging mouse muscle injury sites that is a co-expression of Cdkn2a and Cdkn1a. An interaction between the single-cell senescence scoring system showed that half of the high-scoring cells had this co-expression pattern at a score of 2,412, which is a good quantitative measure to measure muscle aging (Walter et al., 2024). This finding opens the door to developing therapeutic strategies that specifically target and eliminate these senescent cells to improve regeneration in aged muscles. Moreover, studies of human muscle stem cells revealed that accumulating inflammatory conditions cause activated stem cells to abnormally express inflammatory markers, such as CCL2, CXCL1, and IL32, as well as the receptor TNFRSF12/FN14 (De Micheli et al., 2020), which could be targeted as therapy to address muscle dysfunction.

Beyond regenerative medicine, the combination of scRNA-seq and ST is enhancing our ability to diagnose and predict disease progression. The combination of scRNA-seq and ST has greatly increased the ability of biomarkers to predict (Xiao et al., 2024). scRNA-seq has been used in lung transplantation studies to determine the transcriptomic signature in bronchoalveolar lavage fluid that progress before clinical symptoms in chronic allograft dysfunction in patients with lung allograft failure (Brunet-Ratnasingham et al., 2025). While not muscle-specific, this highlights the power of these technologies for non-invasive disease monitoring, a concept directly applicable to tracking muscle dystrophy progression or response to therapy. Research on oral squamous cell carcinoma and non-small cell lung cancer also established that the patterns of gene expression of particular spatial ecotypes can be used as efficient predictors of patient survival outcomes (Gulati et al., 2025). The spatial interaction patterns between PD-1^+^ T cells and PD-L1^+^ myeloid cells have been analyzed and have provided a new molecular framework used to predict immunotherapy response (Gulati et al., 2025).

Studies on muscle development regulators have also registered considerable advancement. The levels of TCF7L2, an outcome of Wnt signaling pathway, directly affect the lineage commitment of FAPs, where high activity levels stimulate adipogenic differentiation of FAPs (Dell’Orso et al., 2019). The expression of sarcomeric structural proteins, e.g., cTnT and alpha-actinin, are an objective parameter of assessing the efficiency of reprogramming in the studies of cardiac reprogramming (Ran et al., 2017). It is important to note that following 4 weeks of induction using certain reprogramming combinations, more than 60 percent of cells co-expressed the structural proteins, and acquired regular sarcomeric structures (Ran et al., 2017). This observation offers a significant foundation on which the effectiveness of cardiac regeneration therapies can be evaluated.

### 6.2 Agricultural applications in animal breeding and cultivated meat

The cellular and spatial principles elucidated by scRNA-seq and ST hold tremendous promise for revolutionizing animal breeding by moving from tissue-level to cell-level understanding of complex traits. The identification of key cell subpopulations, such as specific MuSCs with high proliferative potential or FAPs predisposed to adipogenesis, provides a cellular basis for precision breeding. For instance, the discovery of CAV1+ MuSCs with high engraftment potential (Barruet et al., 2020) could inspire strategies for *in vitro* muscle stem cell expansion and selection to enhance lean tissue growth. In pigs, the activity of PAX7+ MuSCs in the early postnatal period is a key determinant of final myofiber number, a trait fixed at birth and directly correlated with adult muscle mass. Furthermore, the construction of spatiotemporal atlases for agricultural animals like cattle (Cai et al., 2023a) and chicken (Li et al., 2025) directly maps the dynamic expression of genes controlling muscle fiber type (affecting meat tenderness) and density. For example, the ratio of MYH1 (fast-glycolytic) to MYH7 (slow-oxidative) fibers, resolvable by ST, directly influences meat texture and water-holding capacity in beef and pork. Breeders can leverage these atlases to identify key regulatory cells and genes for marker-assisted selection, genomic selection, or even precision gene editing, aiming to steer the developmental trajectory of muscle tissue towards superior meat yield and quality. Table 6 summarizes key genes and cell populations with potential in livestock breeding. Ultimately, integrating single-cell and spatial multi-omics data into genomic prediction models is expected to significantly improve the accuracy of breeding value estimation for complex traits such as growth rate and carcass composition. Furthermore, the burgeoning field of cultivated meat (cell-based meat) production stands to benefit immensely from the insights derived from scRNA-seq and ST. The primary challenge in cultivated meat is to efficiently proliferate and differentiate MuSCs or other myogenic progenitors at an industrial scale to recapitulate the texture and nutritional profile of conventional meat. ScRNA-seq has been instrumental in defining the optimal cell states for differentiation, identifying markers for highly proliferative “reserve cells” (Benny et al., 2022) and revealing the signaling pathways (e.g., MEK/ERK, NOTCH) that must be modulated to drive efficient myogenic commitment and fusion (Song et al., 2023). ST can further guide the engineering of 3D scaffolds and bioreactor microenvironments by mapping the spatial organization of fibers, ECM, and niche cells in native tissue. By mimicking the spatially patterned cues identified through these technologies, cultivated meat producers can create products with greater architectural and textural fidelity to farm-raised meat.

**TABLE 6:** Key genes and cell populations with breeding potential in livestock.

Species	Key gene/Cell population	Associated trait(s)	Breeding potential/Application
Cattle	CAV1+ MuSCs (Barruet et al., 2020)	Muscle stem cell engraftment, lean growth	Selection for stem cells with high regenerative capacity; *in vitro* meat production
	*MYH1*/*MYH7* Fiber Ratio (Ruoss et al., 2022)	Meat tenderness, metabolic type	ST can map fiber-type spatial distribution for selective breeding towards desired meat quality
Pig	IGF2 (Epigenetic status)	Muscle mass, lean meat percentage	Classic example of a paternally imprinted gene; its methylation status is a key epigenetic marker for selection
	PAX7+ MuSCs (Early postnatal)	Myofiber number, potential for muscle hypertrophy	Early-life marker for ultimate muscle mass potential
	FAPs with high adipogenic potential (e.g., *TCF7L2* activity) (Dell’Orso et al., 2019)	Intramuscular Fat (IMF)/Marbling	Balancing selection to optimize IMF without excessive backfat
Chicken	*MYH1* (Fast-twitch) vs. *MYH7* (Slow-twitch) (Li et al., 2025)	Meat texture, growth rate	Broilers selected for fast growth typically have a higher proportion of *MYH1* fibers
	Daheng vs. Tibetan-specific trajectories (e.g., *SNRPG*, *MYOG*) (Li et al., 2025)	Differentiation rate, muscle composition	Understanding divergent developmental pathways can inform crossbreeding or gene editing strategies

## 7 Challenges and future perspectives

Although scRNA-seq and ST technologies have provided new avenues of research in the area of muscle development, the use of this technology is limited by a number of technical constraints. The main limitation of scRNA-seq is that it has low mRNA capture, and the existing technologies have 10–20 per cent reverse transcription of mRNA molecules (Stegle et al., 2015). This ineffective conversion is what leads straight to the paucity of gene expression information. Despite the fact that ST can retain invaluable spatial data, the resolution-throughput trade-off is one of the issues that persist (Moor and Itzkovitz, 2017; Wan et al., 2023). The problem lies especially with technical artifacts that are added in the process of sample preparation and which may distort transcriptional profiles or result in the loss of certain cell subpopulations (Brunet-Ratnasingham et al., 2025). A particularly critical and muscle-specific challenge is that standard scRNA-seq workflows, which rely on dissociating fresh tissue into single-cell suspensions, fail to capture the multinucleated myofibers that constitute the bulk of muscle tissue. These myofibers are too large and fragile to survive the dissociation process, meaning scRNA-seq inherently samples only the mononuclear interstitial and stem cell populations. Consequently, the transcriptomic state of the myofiber itself, including its specialized myonuclear subtypes, is almost entirely lost. While snRNA-seq on fixed or frozen tissue can partially overcome this by isolating single nuclei from myofibers, it introduces its own biases, such as underrepresentation of certain RNA species and the inability to distinguish between truly multinucleated syncytia and clustered single cells. The irreconcilable nature of multinucleated myotube architecture of skeletal muscle and microfluidic chip-based systems (Zeng et al., 2016) only increases the risk of incomplete cellular representation.

Difficulties in analyzing data are also the issues that need a serious consideration. There is a special need in definition of neighborhood due to the sparsity of ST data, as well as the large cellular heterogeneity (Yuan and Bar-Joseph, 2020). Existing techniques of combining single-cell and spatial data are underdeveloped, particularly in cases of data reconciliation between data that were obtained through various technological platforms (Wan et al., 2023). Despite a large variety of different batch effect correction algorithms, it is difficult to differentiate between biological and technical signals when working with high-dimensional and highly sparse data of scRNA-seq (Tischler and Surani, 2012).

The optimization of integrative multi-omics analytical frameworks will play the central role in the further development of research. The priorities here are to refine the Transformer architectures that are able to model the long-range cellular dependencies in ST data (Liu et al., 2025) and to create new algorithms that are able to harmonize multi-dimensional datasets that have scRNA-seq, epigenomic, and proteomic profiles. Future muscle research will greatly benefit from technologies that enable simultaneous measurement of gene expression and spatial positioning within the same intact myofiber, as well as computational methods designed to integrate snRNA-seq data from myonuclei with scRNA-seq data from interstitial cells. At the same time, it is important to set up a system of standardized analytical pipelines and data-sharing platforms to assure the reliability and reproducibility of research findings (Fu et al., 2025). Also of significance is improving the resolution of ST technologies, which requires advancement of new technologies that are able to measure gene expression and spatial positioning information simultaneously to better solve the spatial-temporal dynamics of interactions among cells in muscle tissue.

## 8 Conclusion

The recent development of scRNA-seq and ST has significantly improved the comprehension of the regulation behind the development of muscles on a molecular level. Such technologies have already managed to produce high-resolution cellular atlases between embryonic and adult developmental periods, with revealing the cellular heterogeneity and dynamic transitions; revealed the complex regulatory networks in which important transcription factors, signaling pathways and epigenetic modifications contribute to fate and tissue patterning; and provided profound understanding of the pathological mechanisms of various muscular diseases. Despite these difficulties in sample preparation, the limitation to resolution, the incorporation of data, and cost-effectiveness, the fast-paced technological advancements, especially higher-resolution spatial platforms, multimodal integration, and AI-driven analytics will continue to drive the field. The study of dynamic processes, functional genetic validation and clinical translation are going to be more and more examined in future studies. Simultaneously, using the technologies on major livestock species will play a significant role in the deconvolution of the cellular and spatial foundation of production characters. It is hoped that the combination of single-cell and spatial multi-omics data to genomic prediction models will improve the accuracy of the breeding value estimation that will eventually provide a breakthrough in the next innovation in the field of breeding animals more precisely and in environmentally friendly meat production.

## Nomenclature

ECM: extracellular matrix
scRNA-seq: single-cell RNA sequencing
ST: spatial transcriptomics
MPCs: myogenic progenitor cells
MuSCs: muscle satellite cells
snRNA-seq: single-nucleus RNA sequencing
scATAC-seq: single-cell Assay for Transposase-Accessible Chromatin using sequencing
MRFs: myogenic regulatory factors
EOM: Extraocular muscles
TA: tibialis anterior
**CAV1** ^**+**^: Caveolin-1
FAP: fibro-adipogenic progenitor
EM: early muscle
LM: late muscle
PSC: pluripotent stem cell
MRFs: myogenic regulatory factors
EEE: early epaxial enhancer
AAV: adeno-associated virus
SR: sarcoplasmic reticulum
NICD: notch intracellular domain
NPCs: neural progenitor cells
PGE2: Prostaglandin E2
TTP: tristetraprolin
IMF: intramuscular fat
FSHD: facioscapulohumeral muscular dystrophy
DMD: Duchenne muscular dystrophy
MYH8: myosin heavy chain
MYL4: myosin light chain 4
DGC: dystrophin-glycoprotein complex
